# The *Acinetobacter baumannii* K70 and K9 capsular polysaccharides consist of related K-units linked by the same Wzy polymerase and cleaved by the same phage depolymerases

**DOI:** 10.1128/spectrum.03025-23

**Published:** 2023-11-17

**Authors:** Anastasiya A. Kasimova, Nowshin S. Sharar, Stephanie J. Ambrose, Yuriy A. Knirel, Mikhail M. Shneider, Olga Y. Timoshina, Anastasiya V. Popova, Andrey V. Perepelov, Andrey S. Dmitrenok, Li Yang Hsu, Ruth M. Hall, Johanna J. Kenyon

**Affiliations:** 1 N. D. Zelinsky Institute of Organic Chemistry, Russian Academy of Sciences, Moscow, Russia; 2 Centre for Immunology and Infection Control, School of Biomedical Sciences, Faculty of Health, Queensland University of Technology, Brisbane, Australia; 3 School of Life and Environmental Sciences, Faculty of Science, University of Sydney, Sydney, Australia; 4 M. M. Shemyakin and Y. A. Ovchinnikov Institute of Bioorganic Chemistry, Russian Academy of Sciences, Moscow, Russia; 5 State Research Center for Applied Microbiology and Biotechnology, Obolensk, Moscow Region, Russia; 6 Saw Swee Hock School of Public Health, National University of Singapore, Queenstown, Singapore; 7 Yong Loo Lin School of Medicine, National University of Singapore, Queenstown, Singapore; Emory University School of Medicine, Atlanta, Georgia, USA

**Keywords:** *Acinetobacter baumannii*, capsular polysaccharide, K70, Wzy polymerase, phage depolymerase

## Abstract

The extensively antibiotic-resistant *Acinetobacter baumannii* GC1(ST1^IP^) isolate SGH0807 from Singapore carries the KL70 gene cluster. The structure of the K70 capsular polysaccharide (CPS) produced by SGH0807 was determined using sugar analysis and one- and two-dimensional ^1^H and ^13^C NMR spectroscopy. The K70 CPS consists of branched tetrasaccharide K-units and is closely related to the previously reported K9 CPS. The KL70 and KL9 loci differ in a short segment that encodes the initiating transferase for d-Fuc*p*NAc in K70 and d-Glc*p*NAc in K9. The two structures differ only in the identity of the “first” sugar of the K-unit, d-Fuc*p*NAc in K70 and d-Glc*p*NAc in K9. This difference alters the identity of one of the sugars involved in the linkage between K-units formed by the Wzy polymerase. However, KL70 and KL9 encode an identical Wzy polymerase, designated as Wzy_KL9_, indicating that the differences between d-Fuc*p*NAc and d-Glc*p*NAc do not affect its function. Whether the difference in the first sugars was recognized by the depolymerases encoded by three K9-specific bacteriophages, AM24, BS46, and APK09, that hydrolyze the bond in K9 CPS formed by Wzy_KL9_ was also examined. Purified depolymerases incubated with K70 CPS purified from SGH0807 formed oligosaccharide fragments that were monomers and dimers of the CPS cleaved at the linkage between K-units. As depolymerases encoded by phage determine host specificity by hydrolyzing specific CPS types, these phages could infect and lyse the SGH0807 K70 isolate. *A. baumannii* carrying KL70 were found in Singapore hospitals between 2006 and 2009.

**IMPORTANCE:**

Bacteriophage show promise for the treatment of *Acinetobacter baumannii* infections that resist all therapeutically suitable antibiotics. Many tail-spike depolymerases encoded by phage that are able to degrade *A. baumannii* capsular polysaccharide (CPS) exhibit specificity for the linkage present between K-units that make up CPS polymers. This linkage is formed by a specific Wzy polymerase, and the ability to predict this linkage using sequence-based methods that identify the Wzy at the K locus could assist with the selection of phage for therapy. However, little is known about the specificity of Wzy polymerase enzymes. Here, we describe a Wzy polymerase that can accommodate two different but similar sugars as one of the residues it links and phage depolymerases that can cleave both types of bond that Wzy forms.

## INTRODUCTION

Novel therapeutic strategies are urgently needed to treat and control the spread of infections caused by carbapenem-resistant *Acinetobacter baumannii*, one of the leading causative agents of high morbidity and mortality associated with antibiotic resistance worldwide (1). Lytic bacteriophage (phage) that selectively binds to and lyses infecting bacteria has demonstrated efficacy against highly resistant strains of *A. baumannii* (2 – 5) and shows promise as alternate or adjunct treatments to antibiotics. The specificity of many *A. baumannii* phage is directed by receptor-binding proteins at their tail baseplate, which often bind to structural epitopes in the polymers of repeating oligosaccharide K-units that make up the capsular polysaccharide (CPS) layer on the cell surface (6, 7). While any single isolate produces just one CPS type, even closely related isolates can produce a different CPS. Overall, the species is predicted to produce many structural forms based on the finding of >200 clusters of genes at CPS biosynthesis K locus (KL) in *A. baumannii* genomes (8). Hence, understanding which CPS structures are produced by problem strains is needed to assist with developing a more targeted approach.

*A. baumannii* isolates resistant to last-line carbapenems, which generally warrant bacteriophage treatment, often belong to established, globally disseminated clonal lineages. Global Clone 1 (GC1) and Global Clone 2 (GC2), which consist of isolates typed as sequence type (ST) 1 and ST2 in the Institut Pasteur (IP) multilocus sequence typing (MLST) scheme, respectively, as well as their single-locus variants, predominate, but other clonal lineages such as ST25 and ST79 are also widespread (9). While variation at the K locus has been detected in most overrepresented STs (10 – 14), systematic, detailed characterization of the CPS biosynthesis gene clusters found in a single clone has only been performed for GC1. One of the earliest analyses reported a total of eight distinct KL (KL1, KL4, KL12, KL15, KL17, KL20, KL25, and KL40) among 45 GC1 genomes, which were found distributed across two phylogenetically distinct clades referred to as Lineage 1 (L1) and Lineage 2 (L2) (12). Since this time, expansion of L1 and L2 into multiple sublineages has been described (13, 15), and further 9 KL have been found, raising the total number to 17 KL identified in GC1 genomes to date.

For the majority of these KL, the structure of the corresponding CPS has been determined (Table 1). The K-units include a variety of different monosaccharide residues, in some cases modified by additional pyruvate or alanine substituents, which are joined together by specific glycosidic linkages. In all cases, the sugars predicted to be produced by specific enzymes or sets of enzymes encoded by genes found in these KL have been found in the corresponding CPS. The identity of the initiating transferase (Itr) that begins CPS biosynthesis with the transfer of a specific sugar to the lipid carrier in the inner membrane also reliably predicts the first sugar of the K-unit (16). The number of genes for glycosyltransferases (Gtrs) that form the linkages between the sugar constituents generally predicts the number of sugars in the K-unit and a specific Wzy polymerase joins completed K-units together to form long-chain CPS prior to export to the cell surface (17 – 19).

**TABLE 1:** CPS corresponding to KL identified in *A. baumannii* GC1 genomes

KL type^*a*^	Original reference reporting KL in GC1	CPS type	CPS structure^*b*^	Isolate used for structural determination	Structure reference
KL1/KL107	(11)(10)	K1	→4)-α-d-Glc*p*NAc-6OAc-(1→4)-α-d-Gal*p*NAcA-(1→3)-β-d-Qui*p*NAc4NR’-(1→	AB307-0294	(20)
KL4	(11)	K4	α-d-Gal*p*NAc-4,6-(*R*)-Pyr	D78	(21)
			1		
			↓		
			6		
			→4)-α-d-Gal*p*NAc-(1→4)-α-d-Gal*p*NAcA-(1→3)-α-d-Qui*p*NAc-(1→		
KL12	(12)	K12	α-Aci*p*5Ac7Ac	D36	(22)
			2		
			↓		
			6		
			→3)-α-d-Gal*p*NAc-(1→3)-α-l-Fuc*p*NAc-(1→3)-α-d-Fuc*p*NAc-(1→		
KL13	(15)	K13	α-Aci*p*5Ac7Ac	UMB001^*c*^	(23)
			2		
			↓		
			6		
			→4)-α-d-Gal*p*-(1→3)-α-l-Fuc*p*NAc-(1→3)-α-d-Fuc*p*NAc-(1→		
KL15/KL147	(12)(15)	K15	→3)-α-d-Qui*p*NAc4NAc-(1→4)-β-d-Glc*p*NAc3NAcA-(1→4)-β-d-Glc*p*NAc3NAcA-(1→3)-α-d-Qui*p*NAc4NAc-(1→	LUH5554^*c*^	(24)
KL17/KL18	(12)(25)	K17	d-Ala	G7	(26)
			↓		
			6		
			→4)-α-d-Gal*p*NAcA-(1→4)-α-d-Gal*p*NAcA-(1→3)-β-d-Qui*p*NAc4NAc-(1→		
KL20	(12)	K20	(*R*)-Pyr	A388	(27)
			4,6		
			→2)-β-d-Gal*p*-(1→3)-β-d-Glc*p*NAc-(1→4)-β-d-Glc*p*A-(1→3)-β-d-Qui*p*NAc4NR-(1→		
KL25	(12)	K25	→3)-β-d-Man*p*NAcA-(1→4)-β-d-Man*p*NAcA-(1→3)-α-d-Qui*p*NAc4NR-(1→	AB5075	(28)
KL40^*d*^	(12)(15)	K91	→4)-β-d-Man*p*NAcA-(1→4)-β-d-Man*p*NAcA-(1→3)-α-d-Fuc*p*NAc-(1→	1053^*c*^	(29)
KL42	(10)	K42	α-Pse*p*5Ac7RHb	LUH5550^*c*^	(30)
			2		
			↓		
			4		
			→3)-β-d-Rib*p*-(1→3)-β-d-Gal*p*NAc-(1→		
KL125	(15)	K125	α-d-Gal*p*NAc	MAR13-1452^*c*^	(31)
			1		
			↓		
			3		
			→4)-β-d-Man*p*NAc-(1→4)-α-l-Fuc*p*NAc-(1→3)-α-d-Fuc*p*NAc-(1→		
KL70	(15)	K70	See Fig. 3	SGH0807	This study
KL146	(15)	K146	−^*e*^	−	−

^*a*^ KL pairs are predicted to produce the same structure based on genetic analysis reported in reference (8).

^*b*^ R’ = Ac or (S)-3-hydroxybutanoyl.

^*c*^ Not a GC1 isolate.

^*d*^ KL40 is a variant of KL91 (not found in GC1) and is expected to produce the K91 structure.

^*e*^ Structure not determined.

Of the 17 KL identified in GC1 to date, only two (KL70 and KL147) have no corresponding CPS structure available (Table 1). The KL70 gene cluster was originally found in a set of unreleased draft genomes assembled from the short-read data in NCBI BioProject PRJEB2801 (Kenyon, Holt, Hall, unpublished). However, the sequence had not been described and is currently only available in our released draft genome (GenBank accession number PYDX01000000.1) of isolate SGH0807 recovered in 2008 from a blood sample at the Singapore General Hospital in Singapore. This genome is reported here but was recently included in a GC1 phylogeny (15).

In this study, we report the properties of *A. baumannii* isolate SGH0807 along with the draft genome sequence and describe the features found in the genome that account for the resistance profile. The structure of the K70 CPS produced by SGH0807 is also reported and correlated to the content of the KL70 CPS biosynthesis gene cluster at the K locus. We further determine the structural epitopes in K70 that are important for phage susceptibility.

## RESULTS

### Extensively antibiotic-resistant *A. baumannii* SGH0807 carrying KL70 belongs to GC1

The draft genome sequence of *A. baumannii* SGH0807 (deposited under NCBI accession number PYDX02000000) is the only instance of the KL70 CPS biosynthesis gene cluster that has been identified in *A. baumannii* genomes to date (8). The draft genome belongs to ST1^IP^, and hence, SGH0807 is a GC1 isolate. SGH0807 is resistant to carbapenems, imipenem, meropenem, and doripenem (meropenem MIC > 32) and was found to be also resistant to ampicillin and third-generation cephalosporins (ceftazidime and cefotaxime), quinolones/fluoroquinolones (ciprofloxacin and nalidixic acid), aminoglycosides (streptomycin, spectinomycin, kanamycin, neomycin, amikacin, and gentamicin), and tetracycline and sulfamethoxazole but was susceptible to colistin (MIC = 0.125). The *catA1*, *sul1*, *tetA*(A), *aadA1*, and *aacC1*, *bla*_TEM-1D_ genes were found in contigs that comprised segments of the AbaR island found in the *comM* gene of Lineage 1 (L1) GC1 isolates (12, 13, 32, 33). However, the *aphA1* gene is missing. The *aphA6* gene (amikacin, kanamycin, and neomycin resistance) in Tn*aphA6* and *oxa23* (carbapenem resistance) in AbaR4 are in a potentially conjugative Aci6 plasmid.

Further analysis revealed that SGH0807 belongs to a recently described sublineage of L1 (13) that includes some unique features including OCL3 at the OC locus (OCL) directing biosynthesis of the outer core of the lipooligosaccharide, an additional copy of the chromosomal *ampC* gene in transposon Tn*6168* and recombination patches of defined length that replace the standard GC1 segments carrying the *gyrA* and *parC* genes. Tn*6168* confers resistance to third-generation cephalosporins (34), and the *gyrA* and *parC* alleles present in SGH0807 confer resistance to fluoroquinolones. This sublineage is further subdivided based on the presence of either a copy of AbaR4 in a specific location in the AB0057 sublineage or the presence of a specific integrated phage genome in the A85 sublineage (13). Members of each of these sublineages were also found to carry one or two copies of ISAba1 in sublineage-specific locations. SGH0807 was assigned to the A85 sublineage because it includes the characteristic prophage region. However, it does not carry the A85-specific ISAba1, indicating that it represents an earlier form. Hence, SGH0807 is an extensively resistant isolate that represents an early member of an important sublineage of GC1.

### Organization of KL70

**Fig 1:**
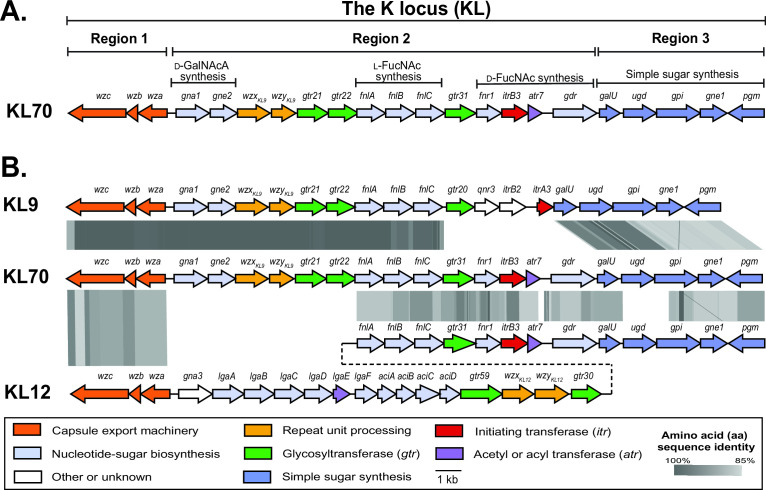
(**A**) Genetic organization of the KL70 gene cluster at the K locus in the *A. baumannii* SGH0807 genome. Figure drawn to scale from GenBank accession number PYDX01000015.1 (base coordinates 31738–61173). The three regions and gene modules involved in sugar biosynthesis are indicated above. (**B**) Comparison of KL70 with *A. baumannii* KL9 and KL12 CPS biosynthesis gene clusters. Genes drawn as arrows are colored according to predicted functions of gene products as indicated by the scheme below. Gray shading indicates regions of >85% amino acid sequence identity.

The specific sequence at the SGH0807 K locus was first identified as KL70 in a previous study (15). However, the genetic content of the locus has not been described in detail. KL70 (annotated and released under GenBank accession number PYDX01000000.1; Fig. 1) has an arrangement typical of most CPS biosynthesis gene clusters identified in *A. baumannii*, in that it includes the three characteristic “regions” (8, 10, 11). Region 1 and region 3 include genes common to most *A. baumannii* KL and consist of *wza-wzb-wzc* genes for capsule export and *galU-pgm* genes for synthesis of simple sugar precursors (e.g., UDP-*N*-acetyl-d-glucosamine; UDP-d-Glc*p*NAc), respectively (11). Genes responsible for the synthesis and processing of the specific K-unit are located in region 2 (8), and for KL70, this region includes three groups of sugar biosynthesis genes. These are *gna1/gne2* genes for synthesis of UDP-*N*-acetyl-d-galactosaminuronic acid (UDP-d-Gal*p*NAcA), *fnlABC* for UDP-*N*-acetyl-l-fucosamine (UDP-l-Fuc*p*NAc), and *fnr1/gdr* for UDP-*N*-acetyl-d-fucosamine (UDP-d-Fuc*p*NAc). The roles of the proteins encoded in these modules were originally predicted via homology to proteins of known function in other species (11). However, these sugars have now been found in all CPS structures from strains carrying these same gene modules (see Table 1 for references).

Region 2 of KL70 is most closely related to region 2 of the KL9 reference sequence found in the complete genome of the GC2 (global clone 2) isolate MDR-TJ (GenBank accession number CP003500.1), which was identified and annotated in a previous study (11). The two gene clusters share 96.93% nucleotide sequence identity over 20,695 bp of the 26,480 bp locus and differ only in a small group of genes in region 2, where *gtr31/fnr1/itrB3/atr7/gdr* in KL70 replace *gtr20/qnr3/itrB2/itrA3* in KL9. The *gtr31/fnr1/itrB3/atr7/gdr* module has previously been described for KL12 and the related KL13, KL73, and KL125 gene clusters (22, 23, 31, 35). Hence, KL70 appears to be a hybrid of these gene clusters. A comparison of KL70 with KL9 and KL12 is shown in Fig. 1B.

As KL70 encodes an ItrB3 initiating transferase that has been shown to initiate K-unit biosynthesis with the transfer of d-Fuc*p*NAc-1-phosphate from UDP-d-Fuc*p*NAc to the lipid carrier in the inner membrane (22, 23, 31), d-Fuc*p*NAc is expected to be the first sugar of the K70 unit. The sequential transfer of sugars onto this d-Fuc*p*NAc residue is carried out by glycosyltransferases, and KL70 carries three glycosyltransferase genes (named *gtr21*, *gtr22*, and *gtr31*) predicting a tetrasaccharide structure. A *wzx* gene and a *wzy* gene, responsible for K-unit translocation and polymerization, respectively, are also present. Hence, the K70 CPS is predicted to be made up of repeating tetrasaccharide units that include d-Gal*p*NAcA, l-Fuc*p*NAc, and d-Fuc*p*NAc residues and is expected to include features found in K9 combined with features found in K12 (K13, K73, and KL125).

### Structural elucidation of the K70 CPS from SGH0807

CPS was isolated from cells of *A. baumannii* SGH0807 by phenol-water extraction (36). Sugar analysis using gas liquid chromatography (GLC) of the alditol acetates and acetylated octyl glycosides derived from the CPS revealed l-FucNAc and d-FucNAc in the ratios ~2:1 (Fig. S4 to S6). Further studies (see below) showed that the CPS also contains d-GalNAcA. Genetic data (see above) indicated that GalNAcA has the d configuration and was consistent with the presence of both l-FucNAc and d-FucNAc.

**Fig 2:**
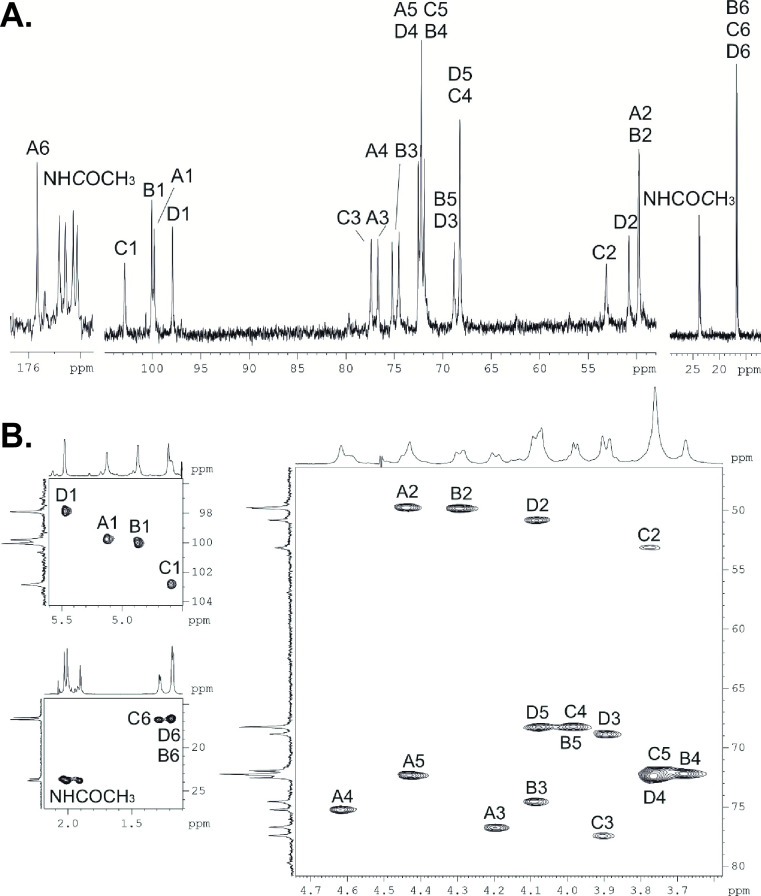
(**A**)^13^C NMR spectrum of the K70 CPS from *A. baumannii* SGH0807. (**B**) ^1^H,^13^C HSQC spectrum of the K70 CPS from *A. baumannii* SGH0807.

The CPS was studied by ^1^H and ^13^C NMR spectroscopy. The NMR spectra of the CPS showed the presence of four sugar spin systems, including those for Gal*p*NAcA (unit **A**) and three Fuc*p*NAc residues (units **B-D**), all monosaccharides being in the pyranose form. In the ^13^C NMR spectrum of the CPS (Fig. 2A; Table 2), there were present, inter-alia, signals for four anomeric carbons at δ_C_ 97.9–102.9, C-6 of GalNAcA at δ_C_ 175.7 (CO_2_H) and FucNAc at δ_C_ 16.5–16.8 (CH_3_), three nitrogen-bearing carbons (C-2) of the amino sugars at δ_C_ 49.8–53.2, and four N-acetyl groups at δ_C_ 23.6–23.8 (CH_3_) and 174.1–174.8 (CO). Accordingly, the ^1^H NMR spectrum of the CPS (Table 2) showed signals for four anomeric protons at δ_H_ 4.59–5.48, H-6 of d-FucNAc at δ_H_ 1.18–1.28 (CH_3_), and four N-acetyl group at δ_H_ 1.91–2.03.

**TABLE 2:** ^13^C and ^1^H NMR chemical shifts (δ, ppm) of the K70 CPS of *A. baumannii* SGH0807 and a modified polysaccharide (MPS) obtained by Smith degradation of the CPS^*a*^

Sugar residue	C-1 H-1	C-2 H-2	C-3 H-3	C-4 H-4	C-5 H-5	C-6 H-6
CPS^*b*^ →3)-α-d-Gal*p*NAcA-(1→ **A**	99.8 *5.13*	49.8 *4.45*	76.8 *4.20*	75.2 *4.63*	72.4 *4.62*	175.7
→3)-α-l-Fuc*p*NAc-(1→ **B**	100.1 *4.88*	49.8 *4.30*	74.6 *4.10*	72.3 *3.68*	68.2 *3.99*	16.5 *1.28*
→3)-β-d-Fuc*p*NAc-(1→ **C**	102.9 *4.59*	53.2 *3.78*	77.5 *3.91*	68.3 *3.99*	*72.4* *3.76*	16.8 *1.19*
α-l-Fuc*p*NAc-(1→ **D**	97.9 *5.48*	50.8 *4.09*	68.9 *3.91*	72.4 *3.76*	68.3 *4.09*	16.5 *1.19*
MPS^*c*^ →3)-α-d-Gal*p*NAcA-(1→ **A**	100.0 *5.07*	49.3 *4.27*	77.0 *4.04*	70.9 *4.54*	72.1 *4.44*	n.f.
→3)-α-l-Fuc*p*NAc-(1→ **B**	100.0 *4.92*	49.9 *4.29*	74.9 *4.05*	71.9 *3.72*	68.4 *4.07*	16.6 *1.19*
→3)-β-d-Fuc*p*NAc-(1→ **C**	103.4 *4.53*	53.1 *3.92*	77.2 *3.82*	68.4 *4.07*	*72.3* *3.78*	16.9 *1.26*

^*a*^ ^1^H NMR chemical shifts are italicized.

^*b*^ The following are the chemical shifts for the N-acetyl groups: δH 1.91–2.03 (Me); δC 23.6–23.8(Me) and 174.1–174.8 (CO).

^*c*^ The following are the chemical shifts for the N-acetyl groups: δH 1.91–2.08 (Me); δC 23.8–23.9(Me) and 174.7–175.1 (CO)^b^.

The ^1^H,^1^H TOCSY spectrum of the CPS showed H1/H2–H4 correlations for spin systems of units **C** and **D** and H1/H2–H3 correlations for units **A** and **B**, which were assigned using the ^1^H,^1^H COSY spectrum (Table 2). A relatively large *J*_1,2_ coupling constant of ~7 Hz indicated that unit **C** is β-linked, whereas the α-linked units **A**, **B**, and **D** were characterized by significantly smaller *J*_1,2_ values (<4 Hz). With the ^1^H NMR signals assigned, the ^13^C NMR spectrum of the CPS was assigned using a ^1^H,^13^C HSQC experiment (Fig. 2B; Table 2).

In the ^13^C NMR spectrum of the CPS, relatively low-field positions of the signals for C-3 of units **A**, **B**, and **C** at δ_C_ 74.6–77.5 and C-4 of unit **A** at δ_C_ 75.2, as compared with their positions in the corresponding non-substituted monosaccharides (37), showed that units **A–C** are 3-substituted, and unit **A** also is 4-substitued. The ^13^C NMR chemical shifts of unit **D** were typical of the non-substituted α-Fuc*p*NAc (37), and hence, this residue occupied the terminal position in the side chain.

The ^1^H,^1^H ROESY (Fig. S1) spectrum of the CPS demonstrated the following correlations between the anomeric protons and protons at the linkage carbons: **A** H-1/**B** H-3, **B** H-1/**C** H-3, **C** H-1/**A** H-3, and **D** H-1/**A** H-4. The chemical shifts of δ_C_ 99.8 and 100.1 for C-1 of units **A** and **B**, respectively, indicated that in the disaccharide fragments **A**-(1→3)-**B** and **B**-(1→3)-**C**, the constituent monosaccharides have different absolute configurations (37, 38). Therefore, unit **A** (GalNAcA) has the d configuration (see above), unit **B** has the l configuration, and unit **C** has the d configuration. As l-FucNAc and d-FucNAc are present in the ratio ~2:1, these data also showed that unit **D** has the l configuration.

**Fig 3:**
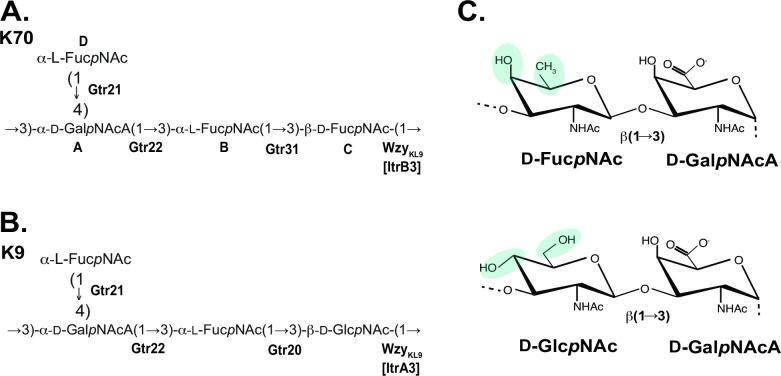
Comparison of CPS and sugar structures. (**A**) Structure of K70 CPS from *A. baumannii* SGH0807 (this work). (**B**) Structure of K9 CPS from *A. baumannii* LUH3484 (39). Glycosyltransferases are shown in bold next to the linkage they have been assigned to. (**C**) Representation of the glycosidic linkages formed by Wzy_KL9_ shown to the right of each CPS structure with differences highlighted by colored shading.

The CPS structure was confirmed by Smith degradation, resulting in a modified polysaccharide that corresponds to the main chain of the K70 CPS. The MPS was studied by ^1^H and ^13^C NMR spectroscopy including two-dimensional experiments ^1^H,^1^H COSY, ^1^H,^1^H ROESY, ^1^H,^1^H TOCSY, and ^1^H,^13^ C HSQC (Fig. S2). Therefore, the CPS has the structure shown in Fig. 3.

### Configuration of the K70 unit and assignment of the first sugar

The K70 CPS is composed of tetrasaccharide units that consist of an α-d-Gal*p*NAcA-(1→3)-α-l-Fuc*p*NAc-(1→3)-β-d-Fuc*p*NAc trisaccharide main chain and an l-Fuc*p*NAc side-branch that is α-(1→4) linked to the d-Gal*p*NAcA residue (Fig. 3A). While the structural analysis cannot determine which residue in the main chain represents the first sugar of the K unit, the presence of *itrB3* in KL70 indicates that d-Fuc*p*NAc is the first sugar (see above). As only one d-Fuc*p*NAc residue is present in the structure obtained, this residue was assigned as the first sugar (as drawn in Fig. 3A).

### Assignment of the Gtrs to linkages in the K70 unit

Gtr31 (GenPept accession number WKC12490.1) is encoded by a gene that has previously been found in a number of *A. baumannii* CPS biosynthesis gene clusters, including KL12 (22), KL13 and KL73 (23), and KL125 (31) from isolates for which the corresponding CPS structures have been determined (see Table 1). In these structures, Gtr31 was predicted to form a shared α-l-Fuc*p*NAc-(1→3)-d-Fuc*p*NAc linkage, and the same linkage is present in K70. In each of these gene clusters, the *gtr31* gene is part of a gene module that also includes *fnr*1 and *gdr* to synthesize UDP-d-Fuc*p*NAc (11) and *itrB3* for the incorporation of d-Fuc*p*NAc as the first sugar.

The remaining glycosyltransferases, Gtr21 and Gtr22, are encoded by genes that are shared by KL9 (Fig. 1B). A structure for the K9 CPS is available [Fig. 3B; named O5 in (39)]. It was determined from the *A. baumannii* GC2 isolate LUH3484 that carries a gene cluster (GenBank accession number KC526895.2) that is referred to as PSgc5 in (40) and is identical to the previously described KL9 gene cluster (10). The shared α-l-Fuc*p*NAc-(1→4)-α-d-Gal*p*NAcA-(1→3)-l-Fuc*p*NAc segment is therefore produced by enzymes encoded by the genes shared by KL70 and KL9. Gtr22 (GenPept accession number WKC12486.1) shares only 28% identity with its closest relative, Gtr19 from *A. baumannii* KL8, which was previously assigned to the α-d-Gal*p*NAc-(1→3)-l-Fuc*p*NAc linkage in the K8 structure (41). In comparison, Gtr21 (GenPept accession number WKC12485.1) was found to share little to no sequence identity with other known *A. baumannii* glycosyltransferases or those of known or predicted function from other species. Hence, Gtr22 was assigned to a similar α-d-Gal*p*NAcA-(1→3)-l-Fuc*p*NAc linkage in K70, leaving Gtr21 for formation of the remaining α-l-Fuc*p*NAc-(1→4)-d-Gal*p*NAcA linkage. The monosaccharide composition of the K70 unit and the internal linkages are therefore consistent with the genetic content of the KL70 gene cluster. This assignment is also consistent with the assignments made previously for KL9 (PSgc5) in LUH3484 (40).

### KL70 and KL9 encode identical Wzy polymerases

As the Wzy protein links the first sugar of a K-unit to another sugar to create the specific configuration of the CPS, the linkage formed by the Wzy polymerase that joins the K70 units would be the β-d-Fuc*p*NAc-(1→3)-d-Gal*p*NAcA. However, the KL70 and KL9 gene clusters encode identical Wzy polymerases [100% coverage and 100% aa sequence identity; annotated as Wzy_KL9_ according to the nomenclature system proposed recently (8)], and the linkage between K9 units is β-d-Glc*p*NAc-(1→3)-d-Gal*p*NAcA. This finding was unexpected, as to date, *A. baumannii* strains with different KL that encode closely related Wzy polymerases (>85% aa identity) have been found to produce CPS with units joined by a linkage that involves the same pair of sugars (17, 23, 27, 42 – 46).

### Wzy_KL9_ likely belongs to the EpsG protein family

To date, Wzy polymerases encoded in *A. baumannii* genomes have belonged to one of three different protein families named EpsG, Wzy_C, and O-ag_pol_Wzy (17, 47 – 49), which are defined by shared hidden Markov models (HMMs). However, Wzy_KL9_, encoded at the K locus, was not assigned to any of the known families or any other protein family established to date, though it shared significant homology with known Wzy. A broader search for Wzy_KL9_ homologs encoded by *A. baumannii* KL revealed that Wzy_KL9_ shares 40% aa identity with Wzy_KL8_, which is known to form a β-d-Glc*p*NAc-(1→3)-d-Gal*p*NAc linkage between units in the *A. baumannii* K8 CPS (41). This linkage is similar to the linkages formed by Wzy_K9_ in the K9 and K70 CPS structures, and this is consistent with the assignment of Wzy_K9_ to formation of those linkages. Though Wzy_KL9_ could not be assigned to a known protein family, Wzy_KL8_ was previously reported as belonging to the EpsG family (41), suggesting that Wzy_KL9_ may also belong to the EpsG family and that the HMM requires adjustment.

### Searches for additional Wzy candidates

As there have been no identified cases in *A. baumannii* of identical Wzy polymerases producing linkages with different first sugars, the possibility that an alternate Wzy is encoded elsewhere in the genome of either SGH0807 or LUH3484 was investigated. Wzy polymerases are integral membrane proteins that exhibit a high level of sequence diversity both within and across bacterial species (50). However, genes located outside the K locus that encode Wzy polymerases can be detected using a combination of simple protein homology-based searches (11). The two ligases responsible for protein or pilin glycosylation in *Acinetobacter* (51) belong to the Wzy_C (PF04932) family but can be easily distinguished.

The coding sequences from both genomes (*n* = 3912 in SGH0807 and *n* = 3690 in LUH3484) were therefore translated and first assessed for HMMs consistent with one of the three known families associated with Wzy. Using *hmmscan* with the currently available Pfam database, one protein from SGH0807 and two from LUH3484 that belonged to Wzy_C were identified as the known protein and pilin ligases. However, none of the other predicted protein sequences from either SGH0807 and LUH3484 genomes were found to belong to EpsG (PF14897) or O-ag_pol_Wzy (PF14296) families.

To ensure that there were no other possible Wzy candidates that did not fall into one of the known families, translated coding sequences were assessed for transmembrane segments (TMS). Those with >7 TMS (excluding the protein and pilin glycosylases and Wzy_KL9_) and no identified protein family (*n* = 4 from SGH0807 and *n* = 5 from LUH3484) were each further subjected to BLASTp to search for homologs of known or predicted function. This returned hits to either permeases, regulatory proteins, or hypothetical proteins found in many *A. baumannii* genomes. Therefore, it was concluded that it is unlikely that any additional Wzy proteins were encoded by either of the SGH0807 or LUH3484 genomes. The lack of an alternate *wzy* gene located elsewhere in either genome indicated that Wzy_KL9_ is able to form similar linkages, even though these linkages involve two different first sugars (β-d-Glc*p*NAc-(1→3)-d-Gal*p*NAcA in K9, and β-d-Fuc*p*NAc-(1→3)-d-Gal*p*NAcA in K70). The chemical structures of d-Fuc*p*NAc and d-Glc*p*NAc are similar (Fig. 3), differing only in hydroxyl groups at carbon C4 and C6. The linkage between units involves only C1 of these sugars, and the differences at C4 and C6 do not appear to influence the function of Wzy.

### Distribution of Wzy_KL9_ and association with other CPS types

The *A. baumannii* KL reference sequence database (8) was screened for further instances of the *wzy*_KL9_ gene to determine its distribution and association with other *A. baumannii* CPS types. The *wzy*_KL9_ gene (100% identity) was found in an additional five KL gene clusters, KL109, KL149, KL168, KL173, and KL222 (Fig. S3A). All but one of these gene clusters include an *itrA3* gene and differ from KL9 and each other only in the sequence located between *gpi/gne1* and *pgm*. So far, genes found in this location have not been found or predicted to have a role in CPS biosynthesis (8). Therefore, strains carrying these loci are expected to produce the K9 CPS type. Equivalent variations on the KL70 gene cluster were not detected.

The remaining gene cluster, KL222, carries an *itrA2* gene replacing *itrA3* but is otherwise closely related to the KL9 group with the *itrA* gene being the only difference between KL222 and KL149 (Fig. S3A). ItrA2 initiates K-unit biosynthesis with the transfer of d-Gal*p*NAc-1-phosphate from UDP-d-Gal*p*NAc to the lipid carrier in the inner membrane (11, 18, 52, 53), and for all *A. baumannii* isolates that produce CPS with d-Gal*p*NAc as the first sugar, an *itrA2* gene is found in the gene cluster at the K locus. The structure of the K222 CPS has not been determined but would be expected to be equivalent to the K9 structure with d-Gal*p*NAc in place of d-Glc*p*NAc as the first sugar. d-Gal*p*NAc differs from d-Glc*p*NAc in the epimerization of the hydroxyl group at carbon C4, and similar to d-Fuc*p*NAc, this carbon is not involved in the linkage between units.

### Influence of the first sugar on phage depolymerase activity

**Fig 4:**
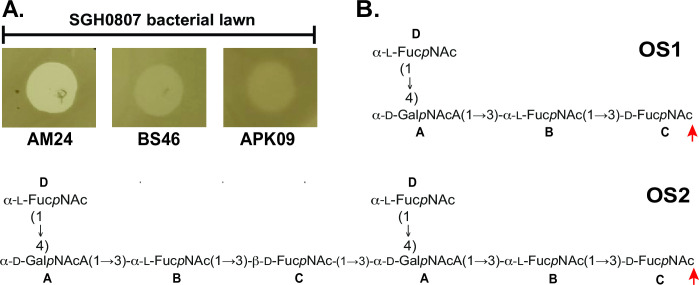
Bacteriophage and depolymerase activity on K70 CPS. (**A**) K9-specific AM24 (54), BS46 (7, 55), and APK09 (56) phage zones of clearing on a lawn of *A. baumannii* SGH0807. (**B**) Oligosaccharide fragments OS1 and OS2 produced by cleavage of K70 CPS from *A. baumannii* SGH0807 by recombinant depolymerase (Dpo) from AM24 and APK09. Red arrows indicate cleavage site.

To determine whether the difference in first sugar influences the ability of phage to recognize, bind, and digest the CPS, the activity of three different bacteriophages, AM24 (54), BS46 (7, 55), and APK09 (56), previously shown to infect only K9-producing *A. baumannii* isolates among a panel of diverse K types, was tested against SGH0807. All three K9-specific phages were found to form zones of clearing on the bacterial lawn of *A. baumannii* SGH0807 (Fig. 4A). This indicated that the difference in the first sugar of the CPS unit did not influence susceptibility of *A. baumannii* SGH0807 to phages AM24, BS46, and APK09.

While none of the resulting zones of clearing formed by the phage had visible halos surrounding them suggestive of depolymerase activity (6, 57), all three phages have been previously reported to encode a Dpo enzyme (7, 54 – 56) with the Dpo from BS46 (DpoBS46) having been demonstrated to hydrolyze the K9 CPS via cleavage of the specific linkage formed by Wzy_KL9_ (7). DpoBS46 (BS46_gp47, GenPept accession number QEP53229.1) shares 73.9% aa sequence identity with DpoAM24 (AM24_gp50, GenPept accession number APD20249.1) over 622 of 842 aa of the protein sequence, with greater homology (92%) over the last 609 aa, which is the region responsible for CPS substrate recognition. In comparison, DpoAPK09 (APK09_gp48, GenPept accession number UAW09804.1) shares only 27.8% and 28.6% aa identity with DpoBS46 and DpoAM24, respectively.

Purified K70 CPS was therefore treated with the previously reported recombinant depolymerases from AM24 (54) and APK09 (56) to determine whether DpoAM24 and DpoAPK09 could hydrolyze the K70 CPS. As the C-terminal region responsible for CPS recognition is highly similar in DpoAM24 and DpoBS46, digestion with DpoBS46 was not performed. Both Dpo treatments were fractionated by gel permeation chromatography, and the same monomer (OS1) and dimer (OS2) CPS fragments were obtained. The molecular mass of the OS1 monomer and OS2 dimer structures were determined by negative ion mode electrospray ionization mass spectrum, which showed peaks of the [M-H]^−^ ion at *m/z* 795.3147 and 1573.6280 against the calculated values *m/z* 795.3147 and 1573.6273, respectively (Fig. S7 and S8). The ^1^H and ^13^C NMR spectra of the monomer (OS1) and dimer (OS2) were fully assigned by two-dimensional NMR spectroscopy and compared with the data of the corresponding CPS to confirm the sequence of the monosaccharides and the presence of a d-Fuc*p*NAc (unit **C**) residue at the reducing end of both structures (Fig. 4B). These data demonstrated that the OS1 monomer and OS2 dimer oligosaccharides were products resulting from Dpo cleavage of the β-d-Fuc*p*NAc-(1→3)-d-Gal*p*NAcA linkage in the K70 CPS that is formed by Wzy_KL9_.

### Distribution of KL70 in *A. baumannii* genomes

To determine the distribution of the K70 CPS in *A. baumannii*, 19,638 *A*. *baumannii* genome assemblies available in NCBI (as of 5th May 2023) were searched for any further instances of the KL70 CPS biosynthesis gene cluster. However, this did not identify any further isolates, suggesting that K70 is a rarely occurring CPS type. *A. baumannii* SGH0807 is from a collection of isolates from the hospitals in Singapore for which draft genome assemblies had never been released (see Introduction). Available short-read data for this collection were assembled into contigs and also screened for the presence of the KL70 gene cluster. In addition to SGH0807, KL70 was detected in further 16 assemblies (deposited into BioProject PRJNA992947) from this collection, which are described in Table 3.

**TABLE 3:** Isolates with genomes that carry KL70

Strain name	Hospital^*a*^	Year	Source^*b*^	ST^IP^	OCL	Resistance genes	BioSample
SGH0807 (DB55809)	SGH	2008	Blood	ST1	OCL3	*aacC1, aadA1, bla*_TEM-1D_, *catA1, sul1*, *tetA*(A)*,* in AbaR3-like *aphA6* in Tn*aphA6, oxa23* in Tn*6022* in an RP-T1 (Aci6) plasmid	SAMN08637738
DR27640	SGH	2008	Sputum	ST1	OCL3	*aacC1, aadA1, aphA1, bla*_TEM-1D_, *catA1, sul1*, *tetA*(A)*,* in AbaR3-like *aphA6* in Tn*aphA6, oxa23* in Tn*6022* in an RP-T1 (Aci6) plasmid	SAMN36698768
DB66738	SGH	2008	Blood	ST1	OCL3	*“^c^*	SAMN36698769
SGH0817 (DR24685)	SGH	2008	Sputum (ETT)	ST1	OCL3	*“*	SAMN36698770
SGH0706 (DR8067)	SGH	2007	Sputum (ETT)	ST1	OCL3	*“*	SAMN36698771
SGH0808 (DB57641)	SGH	2008	Blood	ST1	OCL3	*“*	SAMN36698772
SGH0816 (DB67404)	SGH	2008	Blood	ST1	OCL3	*“*	SAMN36698773
6120468121	CGH	2006	Sputum	ST1	OCL3	*“*	SAMN36698774
9033202376	CGH	2009	Sputum	ST1	OCL3	*“*	SAMN36698775
SGH 60	SGH	2008	Blood	ST1	OCL3	*“*	SAMN36698776
SGH 59	SGH	2008	Sputum (ETT)	ST1	OCL3	*“*	SAMN36698777
SGH0906 (DR7857)	SGH	2009	Sputum (ETT)	ST1	OCL3	*aacC1, aadA1, aphA1, sul1*, *tetA*(A)*,* in AbaR3-like *aphA6* in Tn*aphA6, oxa23* in Tn*6022* in an RP-T1 (Aci6) plasmid	SAMN36698778
SGH0910 (DB19999)	SGH	2009	Blood	ST1	OCL3	*“*	SAMN36698779
DM11156	SGH	2009		ST1	OCL3	*“*	SAMN36698780
9053255619	CGH	2009	Sputum	ST1	OCL3	*“*	SAMN36698781
SGH 46	SGH	2009	Blood	ST1	OCL3	*“*	SAMN36698782
DR18351-1	SGH	2008	Sputum (ETT)	ST2	OCL1	*sul2, tet(B), strA, strB* in AbGRI1 *armA, catB8, sul1*, *aadA1, mph(E), msr(E), aphA1* in AbGRI3	SAMN36698783

^*a*^ Singapore General Hospital (SGH); Changi General Hospital (CGH).

^*b*^ ETT, endotracheal tube.

^*c*^ The “ symbol indicates “same as above.”

These assemblies were subjected to MLST using the *A. baumannii* Institut Pasteur scheme, which revealed that 15 assemblies, like SGH0807, belong to ST1 (GC1) and one to ST2 (GC2) (Table 3). These isolates were recovered over a 4-year period (2006–2009) at the Singapore General Hospital or Changi General Hospital. The GC1 genomes all shared the properties reported above for SGH0807 except that the *aphA1* gene in Tn*6020* was present in the AbaR in all and six had lost the *catA1* and *bla*_TEM-1D_ antibiotic resistance determinants though their loss does not substantially affect the resistance profile. The single ST2 isolate from 2008 was found to carry *armA*, *catB8*, *sul1*, *aadA1*, *mphE*, *msrE*, and *aphA1* in fragments indicating the presence of an AbGRI3-type island and *sul2*, *tet(B)*, *strA*, and *strB* in an AbGRI1-type island. It had also acquired Tn*6168*, and it is likely that this isolate has acquired both the KL70 locus and Tn*6168* from a GC1 isolate.

## DISCUSSION

In this study, we report the genome and properties of the extensively antibiotic-resistant *A. baumannii* isolates recovered at two hospitals in Singapore that carry the KL70 configuration at the K locus. The majority belonged to Lineage 1 of the major globally disseminated clonal group GC1 and carried a variant form of the AbaR3 antibiotic resistance transposon in *comM*. They are early representatives of an important sublineage of L1 in GC1 (13). However, a single isolate belonged to GC2 and appears to have acquired the KL70 gene cluster from one of the GC1 isolates. As data relating to more recent isolates from Singapore are not available, whether this sublineage continues to circulate in Singapore remains to be established.

The complete chemical structure of the K70 CPS produced by isolate SGH0807 was also determined, adding another structure to the set of CPS types found among isolates belonging to GC1. Like many GC1 CPS (Table 1), the K70 structure is acidic due to the presence of a carboxyl group on the 2-acetamido-2-deoxygalactopyranuronic acid (Gal*p*NAcA) residue. However, the structure is distinguished by the presence of three Fuc*p*NAc residues, two of which are l-configured and the other one d-configured. The K70 structure is similar to the reported K9 CPS from the GC2 isolate LUH3484, differing only in the first sugar of the unit where D-Fuc*p*NAc is present in K70 and d-Glc*p*NAc in K9 (Fig. 3). As this sugar represents the reducing terminus of the unit that is linked to another unit by the Wzy polymerase, the linkage between K70 and K9 units involve different first sugars though they are formed by identical Wzy_KL9_ polymerases.

Within *A. baumannii*, more than 134 Wzy types have been identified to date, defined using an amino acid sequence identity cutoff of 85% (8). In general, CPS polymerized by the same Wzy type have units joined by linkages of the same anomeric configuration that involve the same sugars substituted at the same positions (17, 23, 27, 42 – 46). To the best of our knowledge, Wzy_KL9_ is the first example of a Wzy polymerase in *A. baumannii* that is capable of forming linkages between units that involve one of two different first sugars. These sugars, d-Fuc*p*NAc and d-Glc*p*NAc, differ only in epimerization of the hydroxyl group at carbon C4 and an additional OH group at C6 in d-Glc*p*NAc (Fig. 3). The finding of the *wzy_KL9_* gene in another KL (KL222) that carries *itrA2* predicting d-Gal*p*NAc as the first sugar suggests that Wzy_KL9_ may also be able to accommodate a third sugar, d-Gal*p*NAc, which is similarly a C4 epimer of d-Glc*p*NAc. The C4 position of the first sugar, as well as C6, is not directly involved in the linkage between units (C1); therefore, neither epimerization at C4 nor the presence of a hydroxyl group at C6 appears to influence the activity of the Wzy_KL9_ polymerase.

A potentially similar situation was found with the Wzy_KL8_, the closest relative of Wzy_KL ***9.***_ A search of the *A. baumannii* KL reference sequence database identified the *wzy_KL8_* gene in a further three gene clusters. The KL8 CPS biosynthesis gene cluster carries an *itrA3* gene (Fig. S3B), and KL183 also includes *itrA3*. However, the other two, KL217 and KL234, carry an *itrB3* gene (Fig. S3B). While there are currently no structures available for strains carrying these loci, the presence of either *itrA3* or *itrB3* co-located with *wzy_KL8_* at the K locus suggests that Wzy_KL8_ may also be able to form two linkages involving either d-Fuc*p*NAc or d-Glc*p*NAc.

The difference in first sugar was also shown to have no effect on the ability of AM24, BS46, and APK09 phage to infect and lyse SGH0807 or the ability of the encoded depolymerases to hydrolyze the K70 CPS. As recent studies have reported that many *A. baumannii* bacteriophage Dpos specifically cleave the Wzy linkage in the CPS (6, 17, 58, 59), understanding the breadth of specificity of both Wzy types and Dpos could assist with selection of the most appropriate phage for treatment. While the functional mechanism and specificity of Wzy and Dpo proteins are not well understood, this study contributes to our understanding of enzyme specificity.

Development of a targeted approach to bacteriophage therapy relies in part on the ability to not only detect the specific sequence at the K locus and the presence of any additional CPS genes in the *A. baumannii* genome but also accurately characterize the functional Wzy to determine which linkage in the CPS structure represents the bond between units that is usually cleaved by Dpos. Identification of Wzy coding sequences currently relies on the detection of HMM profiles that define Wzy protein families. However, our analysis has shown that while a protein family domain could not be detected in the sequence of Wzy_KL9_ *,* the protein is closely related to Wzy_KL8_, which belongs to the EpsG family, indicating that Wzy_KL9_ is also an EpsG family member. This suggests that HMM profiles for Wzy polymerases may need to be revised to improve currently available search tools.

## MATERIALS AND METHODS

### Bacterial strain, cultivation, and resistance profiling

*A. baumannii* isolate SGH0807 (also known as DB55809) was recovered from a blood sample at the Singapore General Hospital in 2008. Bacteria were cultivated in LB media overnight; cells were harvested by centrifugation (10,000 × *g*, 20 min) and resuspended with phosphate-buffered saline, acetone was added to 70% vol/vol, and cells were precipitated and dried. Resistance of SGH0807 to antibiotics was determined as described previously (12).

### Whole-genome sequencing

Genomic DNA was extracted from *A. baumannii* SGH0807 as described previously (12). DNA was sequenced on an Illumina MiSeq platform at the Australian Genomic Research Facility. Reads were assembled into contigs using SPAdes *v 3.10* (60), and contigs derived from the AbaR region and from Tn*6168* were linked using PCR to confirm their structure and location. The enhanced draft genome sequence was annotated using Prokka and revised manually in keeping with established annotations for the AbaR region (12, 32). Annotations for KL70 reported here were added, and the OCL1 region was annotated as in the reference for OCL1 (10, 11). An unannotated draft genome had been released previously under PYDX01000000 (BioProject number PRJNA421215, BioSample number SAMN08637738). The enhanced draft genome was uploaded to NCBI under WGS accession number PYDX02000000.

Available short-read data for other *A. baumannii* isolates from Singapore hospitals found in PRJEB2801 were assembled using SPAdes *v 3.10*, and genomes carrying KL70 were identified. Assemblies of these genomes have been released under PRJNA992947 (details listed in Table S1).

The short reads for *A. baumannii* isolate LUH3484 (SRA accession DRR006286) were downloaded and assembled into contigs as described above. The correctly annotated KL9 sequence in the LUH3484 genome is available under GenBank accession number KC526895.2

### Bioinformatics analyses

Multi-locus sequence typing was performed using the mlst tool (available at https://github.com/tseemann/mlst) to determine the ST of isolates using the Instiut Pasteur (IP) scheme (available at https://pubmlst.org/organisms/acinetobacter-baumannii). ResFinder *v. 4.1* (https://cge.cbs.dtu.dk/services/ResFinder/) was used to identify resistance determinants. *Kaptive v. 2.04* was used to detect KL and OCL using the latest iterations of the *A. baumannii* KL reference sequence database that includes 241 KL (8) and OCL reference sequence database that includes 22 OCL (61), respectively.

The KL70 sequence from the SGH0807 genome was extracted, annotated according to the established nomenclature scheme (11), and submitted to GenBank under accession number OQ558830.1. Functions of encoded proteins, including glycosyltransferases and the Wzy polymerase, were predicted based on the homology to products of known or predicted function using BLASTp (62) and correlated to the elucidated K70 structure. To identify *wzy* genes outside the K locus, all coding sequences in the assembled genomes of SGH0807 and LUH3484 were identified and annotated using *Prokka v 1.14.15* (63). Amino acid sequences of encoded proteins were submitted to TMHMM *v 2.0* (64) to detect transmembrane segments and *hmmscan v. 2.41.2* (65) to detect hidden Markov models.

### Isolation of the capsular polysaccharide

Bacterial cells (3.7 g) were extracted with 45% aqueous phenol (70°C, 1 h) (36); the extract was dialyzed without layer separation and freed from insoluble contaminations by centrifugation. The resultant solution was concentrated and treated with cold aq 50% CCl_3_CO_2_H at 0°C for 1 h; after centrifugation, the supernatant was dialyzed against distilled water. The yield of the *A. baumannii* K70 CPS was 11.1% (400 mg). A CPS sample (120 mg) was hydrolyzed with 2% CH_3_CO_2_H (100°C, 2 h). Fractionation of the products by gel-permeation chromatography on a column (56 × 2.5 cm) of Sephadex G-50 Superfine (Healthcare) in 0.05 M pyridinium acetate pH 4.5 as eluent gave a purified CPS sample (47 mg).

### Monosaccharide analysis

A CPS sample (1 mg) was hydrolyzed with 2 M CF_3_CO_2_H (120°C, 2 h), reduced with NaBH_4_ in 1 M NH_4_OH (0.5 mL, 10 mg, 20°C, 1 h), and acetylated by a 1:1 (vol/vol) mixture of pyridine and Ac_2_O (120°C, 2 h). Monosaccharides were analyzed by GLC as the alditol acetates on a Maestro chromatograph (Agilent 7820, Interlab, Russia) equipped with an HP-5 column (0.32 mm × 30 m) using a temperature program of 160°C (1 min) to 290°C at 7°C min^−1^.

The absolute configurations were determined by GLC of the trifluoroacetylated (*S*)−2-octyl ester. A CPS and MPS samples (1 mg) were hydrolyzed with 2 M CF_3_CO_2_H (120°C, 2 h), dissolved in a saturated solution of NaHCO_3_, with constant stirring and adding to the solution Ac_2_O in three portions every 15 min (20 µL, 0°C). The solutions were diluted with water, treated with Amberlite resin IR-120 (Na) (BDH Limited Pool, England), filtered, and evaporated.

Then, 2-octanol (0.1 mL) and trifluoroacetic acid (15 µL) were added; after heating (16 h, 120°C), the mixture was acetylated with a 1:1 (vol/vol) mixture of pyridine and Ac_2_O (120°C, 2 h). The resultant octyl glycosides were analyzed by GLC as the alditol acetates on a Maestro chromatograph (Agilent 7820, Interlab, Russia) equipped with an HP-5 column (0.32 mm × 30 m) using a temperature program of 160°C (1 min) to 290°C at 7°C min^−1^.

### Smith degradation

A CPS sample (20.7 mg) was oxidized with aqueous 0.05 M NaIO_4_ (1.4 mL) at 20°C for 48 h in the dark and reduced with NaBH_4_ (35 mg) at 20°C for 16 h. The excess of NaBH_4_ was destroyed with concentrated CH_3_CO_2_H, the solution was evaporated, and the residue was evaporated with methanol (3 × 1 mL), dissolved in 0.5 mL water, and applied to a column (35 × 2 cm) of TSK-40. The modified polysaccharide was eluted with aqueous CH_3_CO_2_H and hydrolyzed with 2% CH_3_CO_2_H (100°C, 2 h). Fractionation of the products by gel-permeation chromatography on TSK-40 followed by HPLC on a column (108 × 1.2 cm) in water gave a MPS sample (15 mg).

### Phage and depolymerase activity determination

*A. baumannii* phage AM24 was obtained from the State Collection of Pathogenic Microorganisms and Cell Cultures «SCPM-Obolensk» (accession number Ph-106). Phage BS46 was received from the Félix d’Hérelle Reference Centre for Bacterial Viruses at Laval University (Québec, Canada). Phage APK09 was isolated in 2018 and detailed described previously (56).

The activity of DpoBS46 and phages AM24, BS46, and APK09 was analyzed via spot-test assay or modification of a double-layer method (66). For this, 200 µL of bacterial culture *A. baumannii* SGH0807 (OD_600_ = 0.3–0.4) was mixed with 4 mL of soft agar (LB broth supplemented with 0.6% agarose), poured onto the nutrient agar. Then, 10 µL of the phage suspensions [~10^9^ plaque-forming units (PFU) per mL] or 10 µL of depolymerase suspension (1 µg) was spotted on the soft agar lawns and incubated at 37°C for 18–24 h.

### Depolymerization of the CPS by recombinant proteins

Purified CPS was solubilized in 100 mM Tris-HCl pH 8.0 buffer, and purified Dpo proteins were added for digestion (1/100 wt/wt). The reaction mixture was kept at 37°C overnight. CPS digestion products were fractionated by gel permeation chromatography on a XK 16 mm (depth) by 100 cm (height) column (gel layer, 800  mm) (GE Healthcare Life Sciences, Chicago, IL, USA) of Fractogel TSK HW-40S (Toyo Soda, Japan) in 1% acetic acid.

### NMR spectroscopy

Samples were deuterium exchanged by freeze drying from 99.9% D_2_O and then examined as solutions in 99.95% D_2_O. NMR spectra were recorded on a Bruker Avance II 600 MHz spectrometer (Germany) at 60°C. Sodium 3-trimethylsilylpropanoate-2,2,3,3-d_4_ (δ_H_ 0, δ_C_ −1.6) was used as internal reference for calibration. Two-dimensional NMR spectra were obtained using standard Bruker software, and Bruker TopSpin 2.1 program was used to acquire and process the NMR data. A 60-ms MLEV-17 spin-lock time and a 150-ms mixing time were used in TOCSY and ROESY experiments, respectively. A 60-ms delay was used for evolution of long-range couplings to optimize ^1^H,^13^C HMBC experiments for the coupling constant of *J*_H,C_ 8 Hz.

### Mass spectrometry

High-resolution electrospray ionization (HR ESI) mass spectrometry was performed in the negative ion mode using a micrOTOF II instrument (Bruker Daltonics). An oligosaccharide sample (~50 ng L^−1^) was dissolved in a 1:1 (vol/vol) water–acetonitrile mixture and injected with a syringe at a flow rate of 3 µL min^−1^. The capillary entrance voltage was set at 3,200 V and the interface temperature at 180°C. Nitrogen was used as the drying gas. The mass range was from *m/z* 50 to 3,500. Internal calibration was done with ESI Calibrant Solution (Agilent).

## SUPPLEMENTAL MATERIAL

Supplemental figures spectrum.03025-23-s0001.docx Figures S1 to S8.

Table S1 spectrum.03025-23-s0002.docx Genomes carrying KL70.

ASM does not own the copyrights to Supplemental Material that may be linked to, or accessed through, an article. The authors have granted ASM a non-exclusive, world-wide license to publish the Supplemental Material files. Please contact the corresponding author directly for reuse.
