# Supplementary material for: The Acinetobacter baumannii K70 and K9 capsular polysaccharides consist of related K-units linked by the same Wzy polymerase and cleaved by the same phage depolymerases
Source: Microbiol Spectr. 2023 Nov 17;11(6):e03025-23. doi: 10.1128/spectrum.03025-23 (PMC10715181; doi:10.1128/spectrum.03025-23)
Supplement: Supplemental figures — Figures S1 to S8. [file spectrum.03025-23-s0001.docx]

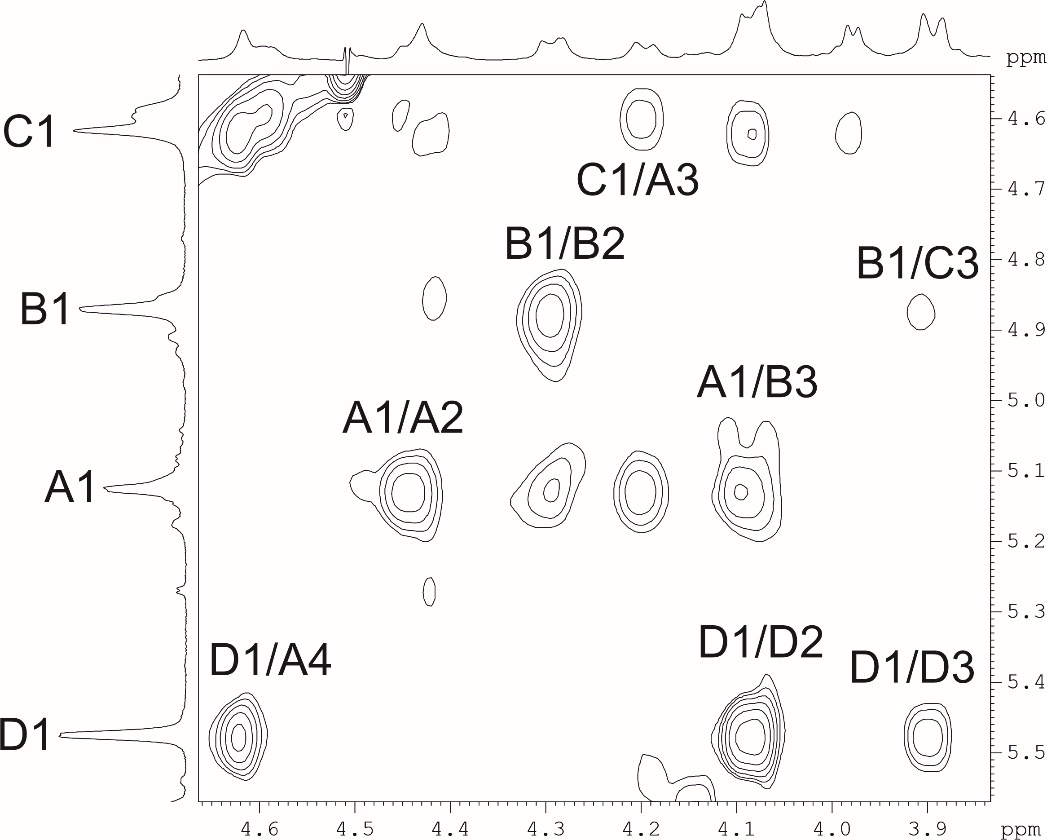


**Figure S1.** Two-dimensional ^1^H, ^1^H ROESY spectrum of the K70 CPS.


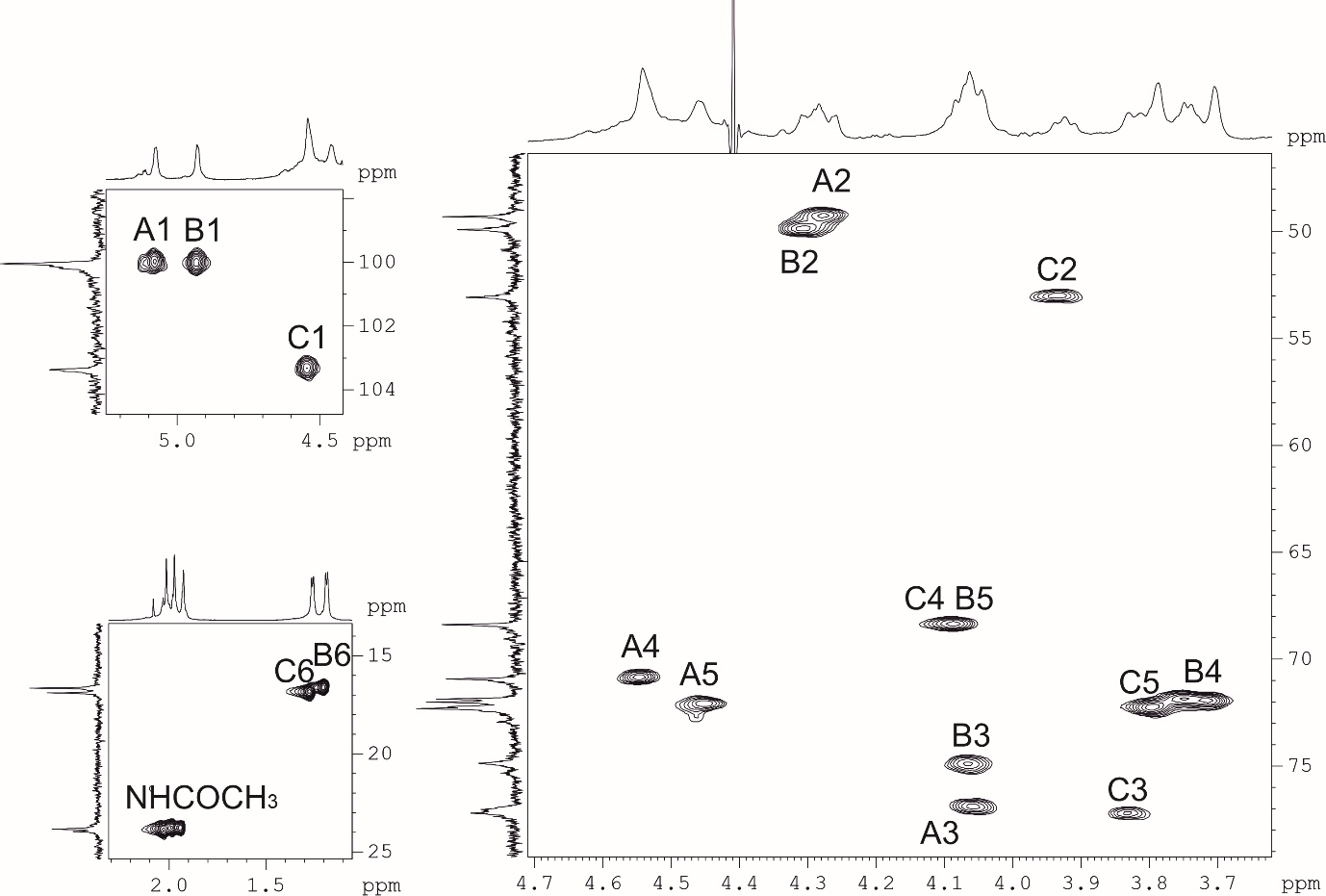


**Figure S2.** ^1^H,^13^C HSQC spectrum of the modified polysaccharide (MPS) obtained by Smith degradation of the K70 CPS.

**Figure S3. Arrangement of *A. baumannii* KL that include a gene coding for Wzy_KL9_ (A) or Wzy_KL8_ (B).** Figure is drawn to scale using sequences available in the *A. baumannii* KL reference sequence database {Cahill, 2022 #523}. Genes drawn as arrows are coloured according to predicted functions of gene products as indicated by the scheme at the top right.

**
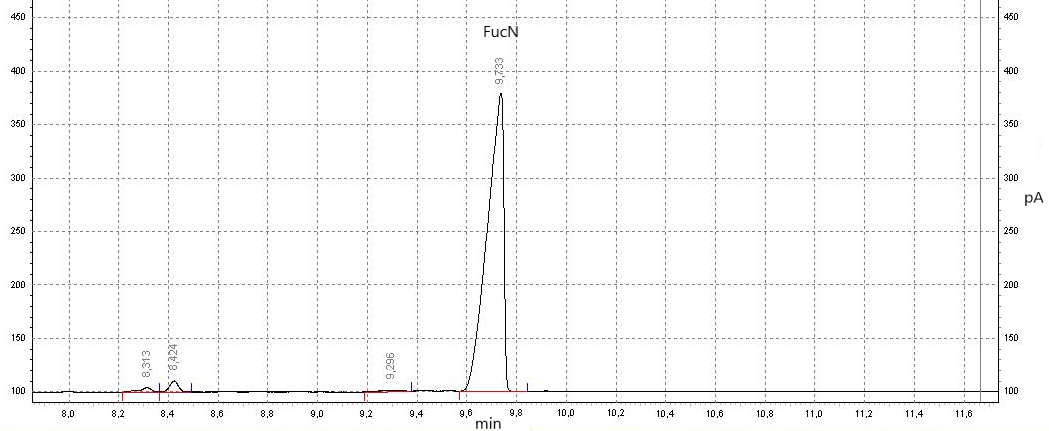
**

**Figure S4. GLC of acetate polyol of FucN from capsular polysaccharide K70.**

**
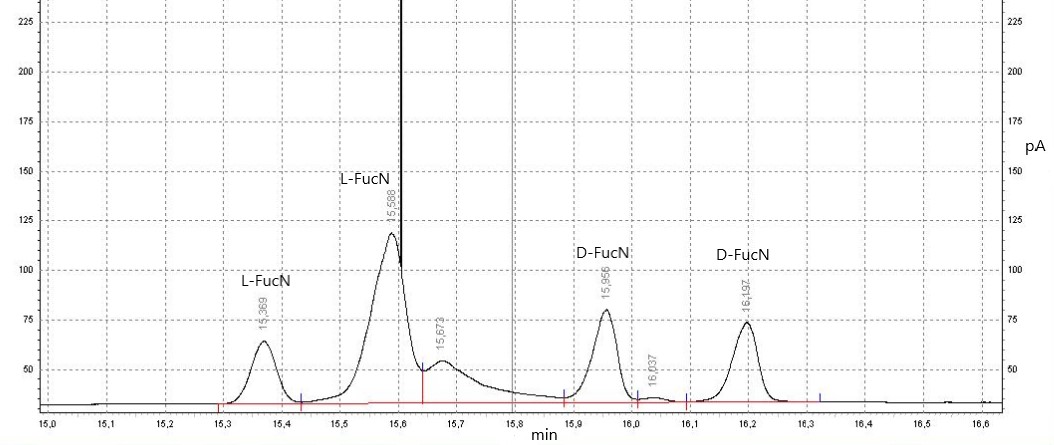
**

**Figure S5. GLC of (+)octyl glycoside of FucN from capsular polysaccharide K70.**

**
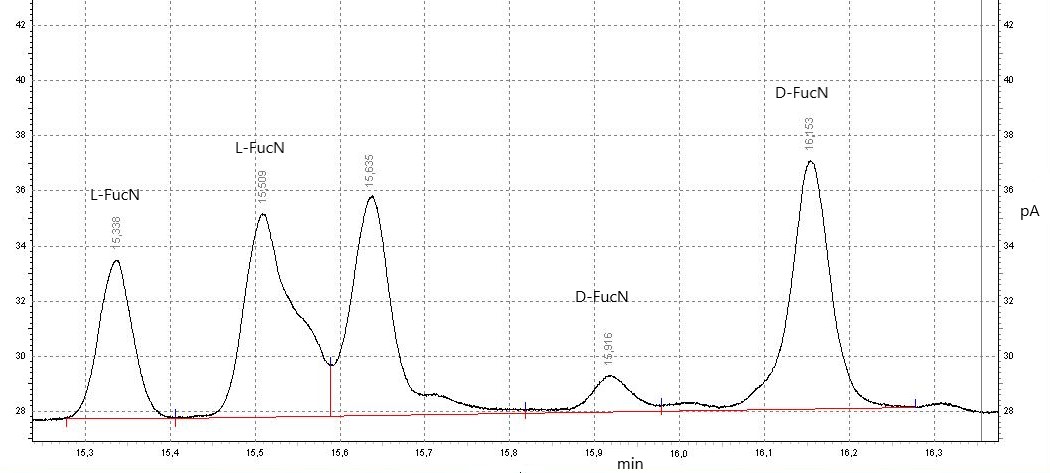
**

**Figure S6. GLC of (+)octyl glycoside of FucN from modified capsular polysaccharide K70 afte smith degradation.**

**
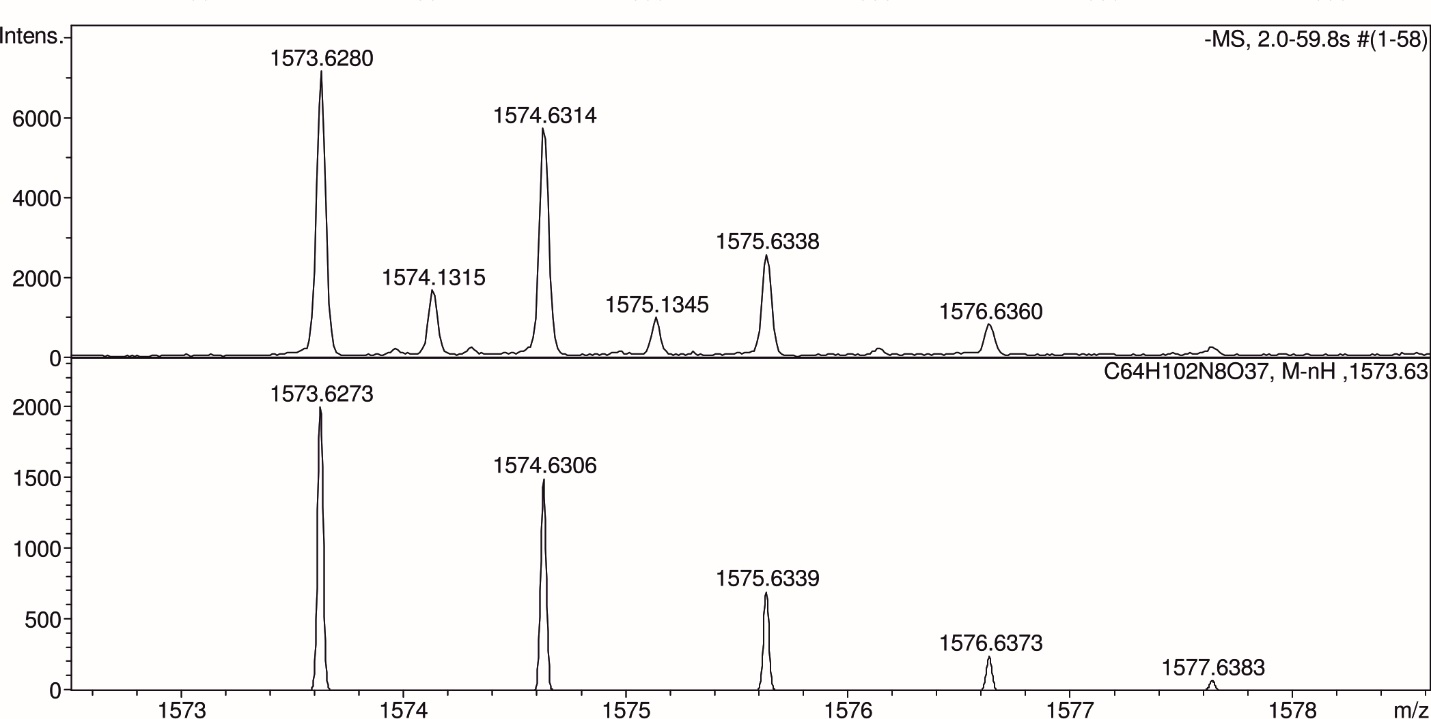
**

**Figure S7. The positive ion mode HR ESI mass spectrum of the OS1.**

**
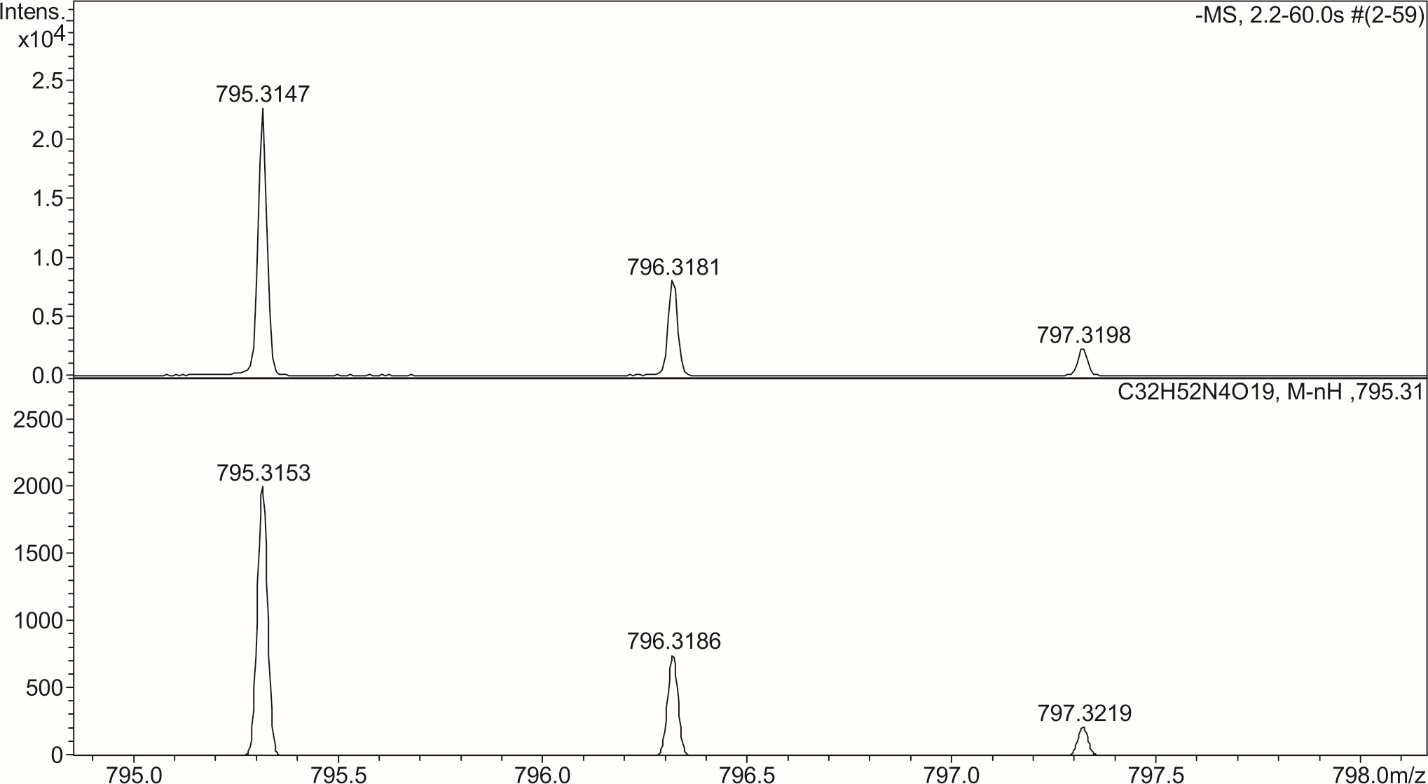
**

**Figure S8. The positive ion mode HR ESI mass spectrum of the OS2.**
