# Supplementary material for: The Acinetobacter baumannii K70 and K9 capsular polysaccharides consist of related K-units linked by the same Wzy polymerase and cleaved by the same phage depolymerases
Source: Microbiol Spectr. 2023 Nov 17;11(6):e03025-23. doi: 10.1128/spectrum.03025-23 (PMC10715181; doi:10.1128/spectrum.03025-23)
Supplement: Table S1 — Genomes carrying KL70. [file spectrum.03025-23-s0002.docx]

**Table S1. Genomes carrying KL70**

|  |  | Read count | Read depth | Number of contigs | Average length of contigs | N50 | BioSample | GenBank Accession |
| --- | --- | --- | --- | --- | --- | --- | --- | --- |
| SGH0807 | DB55809 | 389242 | 14 | 126 | 32870 | 279343 | SAMN08637738 | PYDX0200000 |
| MDRACBA_1b | DR27640 | 1774855 | 67 | 106 | 38747 | 243401 | SAMN36698768 | JAUPHG000000000 |
| MDRACBA_1a | DB66738 | 1983357 | 75 | 105 | 39125 | 279682 | SAMN36698769 | JAUPHF000000000 |
| SGH0817 | DR24685 | 2005718 | 75 | 102 | 40268 | 223268 | SAMN36698770 | JAUPHE000000000 |
| SGH0706 | DR8067 | 2293931 | 86 | 181 | 22881 | 223268 | SAMN36698771 | JAUPHD000000000 |
| SGH0808 | DB57641 | 1799519 | 68 | 160 | 25874 | 208230 | SAMN36698772 | JAUPHC000000000 |
| SGH0816 | DB67404 | 2012433 | 76 | 156 | 26486 | 223268 | SAMN36698773 | JAUPHB000000000 |
| CGH0604 | 6120468121 | 2004936 | 75 | 109 | 37792 | 279682 | SAMN36698774 | JAUPHA000000000 |
| CGH0901 | 9033202376 | 1915315 | 72 | 124 | 33568 | 208230 | SAMN36698775 | JAUPGZ000000000 |
| SGH 60 | MDRACBA_11a | 2034700 | 76 | 104 | 39496 | 243232 | SAMN36698776 | JAUPGY000000000 |
| SGH 59 | MDRACBA_12a | 2019030 | 75 | 244 | 16667 | 223268 | SAMN36698777 | JAUPGX000000000 |
| SGH0906 | DR7857 | 1938314 | 73 | 108 | 38012 | 203508 | SAMN36698778 | JAUPGW000000000 |
| SGH0910 | DB19999 | 1774855 | 67 | 106 | 38740 | 162301 | SAMN36698779 | JAUPGV000000000 |
| MDRACBA_3a | DM11156 | 1764711 | 66 | 115 | 35010 | 156240 | SAMN36698780 | JAUPGU000000000 |
| CGH0904 | 9053255619 | 1915315 | 72 | 111 | 37008 | 171034 | SAMN36698781 | JAUPGT000000000 |
| SGH 46 | MDRACBA_7a | 2144731 | 80 | 101 | 40663 | 203510 | SAMN36698782 | JAUPGS000000000 |
| MDRACBA_4a | DR18351-1 | 2058369 | 77 | 103 | 39575 | 186022 | SAMN36698783 | JAUPGR000000000 |
